# The Peripheral Immune System and Traumatic Brain Injury: Insight into the role of T-helper cells

**DOI:** 10.7150/ijms.46834

**Published:** 2021-09-09

**Authors:** Wangxiao Bao, Yajun Lin, Zuobing Chen

**Affiliations:** Department of Rehabilitation Medicine, First Affiliated Hospital, Zhejiang University School of Medicine, Hangzhou, China.

**Keywords:** traumatic brain injury, cytokines, T-helper cell

## Abstract

Emerging evidence suggests that immune-inflammatory processes are key elements in the physiopathological events associated with traumatic brain injury (TBI). TBI is followed by T-cell-specific immunological changes involving several subsets of T-helper cells and the cytokines they produce; these processes can have opposite effects depending on the disease course and cytokine concentrations. Efforts are underway to identify the T-helper cells and cytokine profiles associated with prognosis. These predictors may eventually serve as effective treatment targets to decrease morbidity and mortality and to improve the management of TBI patients. Here, we review the immunological response to TBI, the possible molecular mechanisms of this response, and therapeutic strategies to address it.

## T cells and traumatic brain injury

Traumatic brain injury (TBI), whose proximate cause is mechanical trauma, is the leading specific cause of death and disability worldwide 1. It is generally accepted that the majority of brain damage caused by TBI is inflicted by secondary effects of the injury, rather than by the primary injury itself 2. Secondary injury, which is progressive and lasts for a long time, contributes significantly to several post-TBI pathological events, including an exacerbated inflammatory response with subsequent brain edema, neuronal apoptosis, and activation of local immune cells, including microglia and astrocytes 3. Additionally, breakdown of the blood-brain barrier (BBB) allows immune cells and molecules to enter the injured brain tissue, where acute and chronic inflammatory reactions to TBI are aggravated 4, 5. Elevated circulating levels of inflammatory cytokines lead to multiple organ dysfunction syndrome and death 6. Immune-inflammatory processes are integral to secondary brain damage 7, in which intracerebral and peripheral immune cells are activated 4, 8 and inflammatory cytokines are recruited 9. Studies TBI models also reveal that TBI can result in immunosuppression. Immune cells, especially lymphocytes, decreased within several hours after TBI, indicating the possible pathophysiological effects 10. The crosstalk between the immune and neurological systems was closely correlated with clinical outcome 11. The presence of concomitant symptoms such as non-neurologic organ injury, neuropsychiatric symptoms and infections make TBI a systematic injury. Ongoing research to reveal post-traumatic immune process may aid in developing effective therapeutic strategies for patients with TBI 12. Sex and age were reported to influence the immune response after TBI. Researchers demonstrate that aged rats exhibited more robust microglial responses, exaggerated secondary neuroinflammation, and worsens neurological outcomes after TBI 13, 14. And TBI leads to a more aggressive neuroinflammatory profile in male compared to female mice, suggesting a rapid and pronounced peripheral inflammatory response and cortical microglia/macrophage activation 15, 16.

Increasing evidence indicates that the immune system is targeted following TBI 17, 18. Neutrophils are first recruited to the site of the damaged brain 19, followed by local activation of microglia and astrocytes as well as the recruitment of other peripheral immune cells, including monocytes, natural killer cells, dendritic cells, and T lymphocytes 20. T lymphocytes, critical constituents of the peripheral immune system, include many subsets, including CD3+, CD4+, and CD8+. In TBI models, CD4+ T cells first increase and then decrease, while CD8+ T cells have the opposite tendency 21, 22. Previous data suggest that autoreactive T cells have beneficial effects on tissue repair following brain injury 23-25. Regarded as T-helper (Th) cells, CD4+ T cells play a central role between antigen presenting cells and B cells. Although Th cells were previously thought to be detrimental 26, several studies have reported a beneficial effect after traumatic injury 27, 28. Evidence shows both potentially destructive (causing autoimmune disease 29) and beneficial (resisting post-traumatic degeneration 30) effects of Th cells in the peripheral immune system after trauma. However, no clear relationship has been established between the levels of T cells and the clinical outcome following TBI.

In this review, we summarize the distinct cellular and molecular events in TBI and highlight the role of Th cells and their cytokines involved in the immune-inflammatory processes associated with brain damage and recovery.

## T cells and their derived cytokines

Th cell subsets, which express CD4 and MHC class II molecules on their surface, begin as naive, uncommitted Th precursors (Th0). Once stimulated by antigen presenting cells, Th cells appear to specifically differentiate into T cell subsets, including Th type 1 cells (Th1), Th type 2 cells (Th2), Th type 17 cells (Th17), and regulatory T (Treg) cells 1, 31. For example, Th0 cells develop into Th1 cells when exposed primarily to interleukin (IL)-12 and interferon (IFN)-γ, whereas they differentiate into Th2 cells when stimulated primarily by IL-4 32. IL-6, transforming growth factor (TGF)-β, and IL-1β are vital factors in Th17 cell development 33 while IL-2 is responsible for Treg cell development 34. Cytokines, a group of messengers released by Th cells, are involved in the subsequent pathophysiological processes that occur in the injured brain 35, 36. Cytokines have pro- and anti-inflammatory effects and play dual roles in secondary brain damage. Both animal and clinical studies have suggested a correlation between TBI and pro- and anti-inflammatory cytokines 37.

Alterations in various T cell subsets as well as their own signature cytokines have been shown to influence immune-inflammatory responses and are associated with the pathogenesis of TBI. Infiltrating T-lymphocytes, cross the BBB via distinct mechanisms, are likely to be associated with brain edema and other acute responses to TBI 38, while activated CD4+T cells may exacerbate the acute damage 39. Studies on the novel immunosuppressive agent FTY720 showed that FTY720 can significantly reduce the number of circulating lymphocytes and attenuate the invasion of immune cells to damaged brain parenchyma 40-42, decrease infiltrating T cells and NK cells but increase the percentage of Treg cells and IL-10 concentration 43. Previous studies have reported the central and peripheral imbalance 44 of Th cells during acute and chronic phases 45 caused by different severities of TBI 21, 46, 47. Many inflammatory mediators in the peripheral immune system have been investigated in TBI patients to identify early biomarkers with diagnostic and prognostic value. Although non-specific inflammatory markers have been extensively studied and reviewed, less attention has been given to the T-cell-specific immunological responses after trauma. Table 1 lists the various T cell subsets and their signature cytokines in the pathogenesis of TBI, including the Th1 cytokines IL-2, IL-12, and IFN-γ, the Th2 cytokines IL-4, IL-5, IL-6, and IL-10, and Th17 and Treg cytokines. However, many of these cytokines are also expressed and released from other cellular sources such as monocytes, microglia, astroglia and neuronal cells 48, which may be reviewed in the future study.

## The Th1/Th2 Balance

The most prominent components of Th cells are the Th1 and Th2 subtypes. Th1 cells are potent activators of macrophages and mediate delayed-type hypersensitivity reactions (also termed cell-mediated immunity), whereas Th2 cells promote antibodies secreted by B cells and immediate-type hypersensitivity reactions (also termed humoral immunity). Cytokines such as IL-2, IL-12, and INF-γ have been characterized as the Th1-associated group of cytokines, whereas cytokines such as IL-4, IL-5, IL-6, and IL-10 have been assigned to the Th2-associated group of cytokines 49.

TBI is accompanied by a severe shift from a Th1- to a Th2-associated response, which may further act as yet-to-be identified risk factor for sepsis, systemic inflammatory response syndrome, and multiple organ failure 50. Shifts in the Th1/Th2 balance also appear in cerebrovascular 51 and neurodegenerative diseases 52, accompanied by various complex interactions and cell signals, suggesting a profound immunological dysfunction. Under normal circumstances, Th0 cells proportionally differentiate into Th1 and Th2 cells. However, a bias toward the Th2 response and Th1 suppression can be induced by TBI 53, which could be associated with a poor clinical outcome 54. Tan et al. 47 reported that administering probiotics improved recovery in TBI patients by adjusting the Th1/Th2 imbalance. The balance between Th1 and Th2 cytokines may be decisive for the progression of TBI. Our discussion will focus on Th1 and Th2 cytokines in peripheral blood.

IL-2 is a pleiotropic cytokine with a complex signaling cascade 55, 56. Among its many actions, IL-2 is a potent Th1 cell growth factor, and an essential factor for the cellular immune response 29. IL-2 is more broadly involved with Th1 57, Th2 58, and Th17 59 cells by regulating the expression of corresponding cytokine receptors 60, 61. Julita et al. 62 demonstrated a significant reduction in serum IL-2 and its soluble receptor (sIL-2R) in TBI patients 10-50 days after trauma, suggesting immunosuppression of IL-2-regulated responses during the post-injury period. He et al. 63 revealed that the serum IL-2/sIL-2R level in trauma patients is low. The decrease of serum IL-2 level and increase of serum sIL-2R level may be involved in the post-traumatic complications and survival, suggesting the prognostic value 64. As an aspect of the cascade of immunological defects after TBI, this decrease in IL-2 may be induced by inhibitory monocytes and immature lymphocytes 65.

In addition to IL-2, IFN-γ, and IL-12 are pro-inflammatory cytokines. IFN-γ is expressed predominantly by Th1 cells, and is an activator of the Th1 immune response and stimulator of IL-12 66. The expression of IFN-γ in the circulating peripheral blood mononuclear cells was thought to decrease in trauma patients because of immune defects 54, but recent evidence suggests that IFN-γ remains persistently high during the acute 67 and chronic phase 68 of TBI. IL-12 had been defined as a promotor of IFN-γ expression and natural killer cell activity 69, 70. IL-12 signaling, related to the development of Th1 71, 72, is governed by the transcription factor signal transducer and activator of transcription 4 through the IL-12 receptor 73. Stahel et al. 74 reported that IL-12 was significantly elevated 14 days after trauma in TBI patients, whereas Schwulst et al. 75 showed that IL-12 expression was subsequently diminished in TBI patients 14 days later. Evidence also shows that peripheral IFN-γ and IL-12 levels are significantly associated with poorer cognitive recovery. Furthermore, high levels of IFN-γ and IL-12 interfere with TBI-induced cognitive impairment 68, 76, thus affecting the magnitude of the behavioral change 76.

IL-4 is an anti-inflammatory Th2 cytokine that downregulates the Th1 response. While it has been generally accepted that Th1 and Th2 cytokines are mutually inhibitory, IL-4 enhances the expression of IL-12 77. Majetschak et al. 78 reported that increased IL-4 levels are more prominent in trauma patients with favorable outcomes than that in those with an unexpected recovery. Kipnis et al. 79 suggested that IL-4 production is induced by T cells after central nervous system (CNS) injury in a MyD88-dependent manner and promotes neuronal survival and recovery through neurotrophic signaling. Although IL-4 levels increase after trauma, they may be protective as well as predictive. Administering IL-4 may be beneficial for patients with TBI by regulating a dysregulated inflammatory response 80, 81.

IL-5 was initially identified to activate B cells, but it exerts pleiotropic functions on various target cells via a high-affinity receptor 82. Trauma patients may exhibit early elevations in plasma IL-5 levels, making them more susceptible to undesirable complications 83, 84.

IL-6 and -10 perform pro- and anti-inflammatory functions, respectively 85, 86 and are contributing factors to the inflammatory response following TBI. In a rat TBI model, IL-6 peaked at 6 h after trauma, while IL-10 peaked at 24 h 35. The IL-6 response is more related to the type of brain damage than the IL-10 response 87. Previous studies have indicated that although increasing IL-6 leads to exaggerated brain damage, IL-6 plays a neuroprotective role by improving post-traumatic healing 88, 89. Kumar et al. 90 reported that elevated IL-6 is associated with an increased inflammatory response, thus leading to an unfavorable global outcome in TBI patients. However, Ley et al. 91 indicated that an IL-6 deficiency in a TBI animal model was associated with poor behavioral performance, suggesting neurotrophic and neuroprotective roles for IL-6. A plasma IL-6 level with a cut-off of 100 pg/mL has been identified to be a predictor for prognosis during the acute phase of brain-injured patients 92.

Apart from a higher pro-inflammatory burden due to IL-6, plasma levels of the anti-inflammatory cytokine IL-10 are significantly higher in TBI patients 93. Elevated serum levels of IL-10 imply a poor outcome after TBI and are positively correlated with injury severity 94, 95. Thus, serum IL-10 at the early phase may have significant prognostic value in TBI patients 96. Administering IL-10 to rat models results in increased neuronal survival by suppressing several inflammatory events 97. Intravenous and subcutaneous, but not intracerebroventricular, administration of IL-10 improves recovery 98. IL-10 also plays an important role in the neuroprotection of hyperbaric oxygen therapy against TBI in mice 99. Contrary to the results from animal experiments, administering IL-10 suppresses the beneficial effects in TBI patients 100. Although IL-10 is consistently elevated during the acute phase of TBI, the contradictory effects of IL-10 occur as a result of different pre-clinical or clinical conditions 101. Kumar et al. 102 reported that an elevated serum IL-6/IL-10 ratio was associated with outcome in TBI patients. The predictive value of IL-6 and -10 in trauma patients remains to be fully elucidated 103.

## The Th17/T-Regulatory cells balance

Th17 cells, characterized by the production of IL-17A, IL-17F, and IL-22, were identified as a new lineage of Th cells in 2005 104. IL-17A is also called IL-17 because it is secreted in the greatest quantities and contributes to most of the Th-17 effects 105. Studies have shown that IL-17 was significantly upregulated after TBI, which may be related to the pathogenesis of TBI 106. As IL-17 is induced with subsequent pro-inflammatory cytokines, Th17 cells have major functions in tissue inflammation. However, recent experiments in Rag1- / - mice have demonstrated that IL-17 is also produced via a RAG-independent cellular source 107. Treg cells are another lineage of Th cells but present a totally different picture compared with Th17 108. Treg cells downregulate the inflammatory response by maintaining peripheral immune tolerance 34, and preventing autoimmunity and chronic inflammation 109. Treg cells are known to be neuroprotective by modulating the function of effector T cells 110 and secreting anti-inflammatory molecules such as IL-10 and TGF-β 111, 112. Similar to IL-10, TGF-β is an anti-inflammatory cytokine that modulates immune-inflammatory processes 113, 114.

The balance between Th17 and Treg cells is critical for the health of the host by controlling inflammatory and autoimmune disorders 33. Besides sharing a similar development pathway, Th17 and Treg trans-differentiate into each other under some conditions 115. A Th17/Treg imbalance has been reported to be associated with severity of injury in trauma patients 116. Gupta et al. have previously showed that the higher the ratio of Th17 cells to Treg, the worse the post-traumatic complications 116. Besides, the imbalance of Th17/Treg cells is believed to be a key factor in the progression of inflammatory response 117, 118. Therefore, adjusting Th17/Treg balance may be an effective way to manage the secondary damage of TBI. Propofol, an intravenous anesthetic drug, maintained Th17/Treg balance and reduced inflammation when injected into the TBI models 119. Emerging findings suggest that the level of circulating Treg cells is positively correlated with neurological recovery in animal models 120 and TBI patients 121. Kipnis et al. 122, 123 reported that transferring exogenous Treg cells into an immune-deficient animal host following CNS injury leads to neuroprotection. Increasing the number of Treg cells and their signature cytokines IL-10 and TGF-β by inhibiting mTOR signaling improves the neurobehavioral performance in brain-injured rats 124.

## Conclusions

Secondary brain injury after trauma is a complex process involving central and peripheral immune responses 4. Immune-inflammatory processes play a vital part in the pathophysiology of TBI. BBB dysfunction allows the passage of immune cells and inflammatory molecules that trigger a systemic inflammatory response 125. Immune-inflammatory processes play a vital part in the pathophysiology of TBI. Recent evidences have established the role of Th cells and their derived cytokines in TBI. Cytokines play a dual role in TBI depending on different time courses and concentrations. A more comprehensive understanding of the cytokines in TBI is needed to develop diagnostic and therapeutic products. Modulating the immunological balance between Th1/Th2, Th17, and Treg cells may also represent a promising therapeutic strategy. Additional investigations are needed to elucidate the basic pathological mechanism of Th cells and their cytokines in the pathogenesis of TBI, and to open up new possible avenues for treating secondary brain injury.

## Figures and Tables

**Figure 1:**
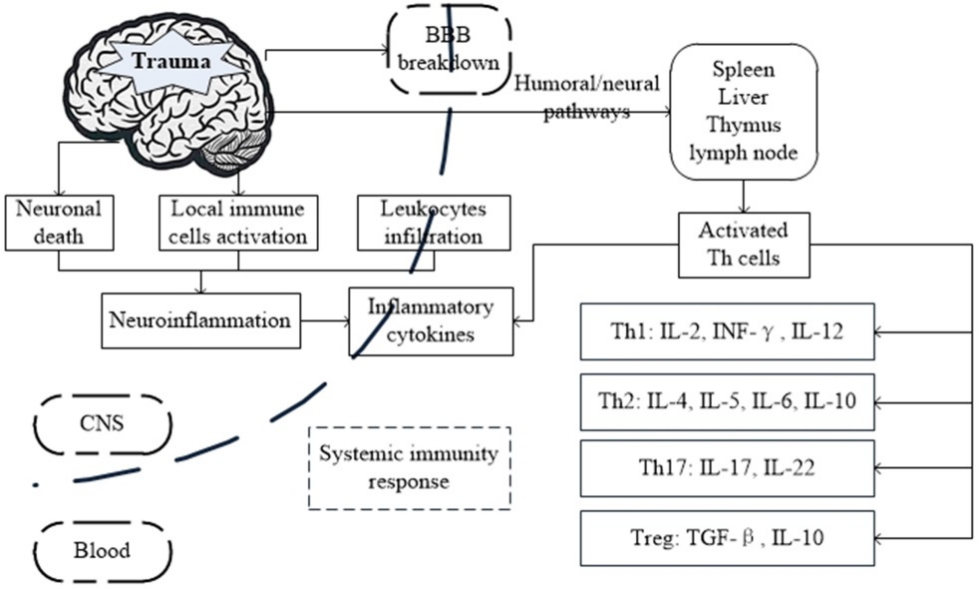
Possible mechanism and the interactions between brain and systemic immunity response after traumatic brain injury (TBI).

**Table 1:** The role and function of T-helper cells and their cytokines in traumatic brain injury

T cell subsets	Cytokines	Peripheral level	Role	Function in TBI
Th 1	IL-2	diminished	Pro-inflammatory	Immunosuppression of IL-2-regulated response in TBI patients
Th 1	INF-γ	elevated	Pro-inflammatory	Interfere with TBI patients' cognitive functioning
Th 1	IL-12	elevated/diminished	Pro-inflammatory	A contributing factor to TBI-induced cognitive impairments in rats
Th 2	IL-4	elevated	Anti-inflammatory	Beneficial for TBI animal models and patients
Th 2	IL-5	elevated	Pleiotropic	Marking TBI patients more susceptible to undesirable complications
Th 2	IL-6	elevated	Pro-inflammatory	Neurotrophic and neuroprotective effects in TBI animal models and patients
Th 2 & Treg	IL-10	elevated	Anti-inflammatory	Beneficial and detrimental effects in TBI animal models
Th 17	IL-17	diminished	Pro-inflammatory	Induce the production and recruitment of pro-inflammatory cytokines after TBI
Treg	TGF-β	elevated	Anti-inflammatory	Improve the neurobehavioral deficits in brain-damaged rats
